# The role of onsite Implanon insertion training for HEWs for sustainable FP programs in Ethiopia: a mixed-method study

**DOI:** 10.1186/s12960-023-00840-6

**Published:** 2023-07-13

**Authors:** Yewondwossen Tilahun, Bekele Belayihun, Luwam Teshome, Habtamu Zerihun, Mengistu Asnake

**Affiliations:** Pathfinder International, Addis Ababa, Ethiopia

**Keywords:** Health extension worker, Onsite-training, Offsite-training, Implanon insertion, Ethiopia

## Abstract

**Background:**

Training health extension workers on Implanon insertion offsite, or away from the workplace, can be cost-intensive, can depend on the human and financial resources of partners, and can compromise routine health services by taking health workers off the job. To address these limitations, the USAID Transform Primary Health Care Activity in Ethiopia designed an onsite Implanon insertion training at the primary health care level. This study compared and documented the implementation experience of onsite vs offsite Implanon insertion training for health extension workers.

**Methods:**

In a mixed-method study conducted in March 2020, the team collected training data from 468 participants—half trained onsite and half offsite—and conducted key informant interviews with 20 purposively sampled individuals. The team analyzed this data, summarizing the data in tables and figures and performing a *t* test with *p* value < 0.05 using SPSS v.20. Qualitative data were analyzed manually in Excel and summarized in Word based on emerging themes.

**Results:**

Health extension workers trained onsite were away from routine work an average of 3 days compared to 8 days for those trained offsite (*P* < 0.001). The difference in average per-trainee cost of onsite (2707 Birr = 87.3 USD) and offsite (6006 Birr = 193.7USD) training was significant (*P* < 0.001). There was no significant difference in mean scores of onsite and offsite trainees on the knowledge pre-test (*P* < 0.947) and post-test (*P* < 0.220) or in simulated practice on an arm model (*p* < 0.202). Onsite trainees, assigned to their own health post for clinical practice, performed Implanon insertions on an average of 10 clients: offsite trainees on an average of 5 clients. Most interview participants reported that the onsite Implanon training was better organized, conducted, followed up, and monitored by health centers to ensure community-level access to Implanon services, with quality and continuity.

**Conclusions:**

Onsite training is a promising approach and minimizes service interruption. It is a likely strategy for on-demand training of health extension workers and immediate assignment of skilled providers to ensure access to and continuity of quality community-level Implanon care.

*Trial registration* N/A.

## Background

Evidence shows that use of contraception is a significant and effective primary prevention strategy to reduce maternal mortality in low- and middle-income countries [1]. Contraceptive implants are being scaled up with a lot of investment, which has dramatically improved their use and made them a popular method everywhere they have been added to the mix of contraceptive methods, but their availability has been constrained due to a shortage of experienced and trained providers [2]. In 2004, Ethiopia introduced its flagship health extension program to provide community-level family planning (FP) services along with other basic health services. Over the past two decades, various organizations have implemented FP interventions in the community through health extension programs in partnership with the Ethiopian government to address the needs of millions of Ethiopian women and girls who wish to delay or limit births [3–6].

Long-acting reversible contraceptives (LARCs) offer very effective protection from unintended pregnancy [7]. Yet, despite increased access to and use of LARCs in Ethiopia, implants account for only 9%, and intrauterine contraceptive devices (IUCD) only 2%, of total modern contraceptive use among all women [8]. To expand access to LARCs, the Ministry of Health (MOH) approved a task-sharing strategy to allow Implanon insertion by health extension workers (HEWs), with support from its partners [3].

Safe, correct, effective insertion and removal of contraceptive implants requires clinical skills and training [2]. To enhance the Implanon insertion skills of HEWs, the MOH designed an offsite in-service training (IST) approach for HEWs in 2009 [3–6]. This entailed significant investment by MOH and development partners in strengthening the capacity of HEWs to provide quality contraceptive services at the community level [9]. However, studies demonstrated that offsite IST is not only cost-intensive but also depends on development partners. Furthermore, it compromises routine health services by removing providers from their workplaces for an extended period [10–12].

Similar studies indicate that the typical in-service training approach (offsite) has some limitations in enhancing and maintaining provider performance, and that trainees were ineffective in putting their training into practice after finishing the training [9]. While in-service training can improve health worker knowledge, skills, behaviors, and attitudes (which are competencies), it may not address the "know-do" gap to improve performance. New evidence suggests that learning within the workplace, in short segments with frequent practice and a focus on doing, rather than knowing, is most effective at impacting performance [10].

The United States Agency for International Development’s (USAID’s) Transform: Primary Health Care Activity, implemented by a consortium led by Pathfinder International in partnership with the Ethiopian MOH, has been working to prevent maternal, child, and neonatal deaths by strengthening the country’s primary health care system at all levels. The Activity supports the attainment of the Health Sector Transformational Plan I and II (HSTP I and II) agendas of the government of Ethiopia. To overcome some of the limitations of offsite training, the Activity designed onsite Implanon insertion training for HEWs at the primary health care unit (PHCU) level. The Activity worked with health centers (HCs) to organize teams of trainers—competent clinical care providers trained on Implanon insertion and removal and working at the PHCU HCs—and provided them with training materials, technical assistance, and financial support to conduct the trainings. This model also promoted sustainability by strengthening the capacity of PHCU-HCs to organize, conduct, and track training themselves by integrating the program into the existing health system, so that newly assigned untrained HEWs in the health posts (HPs) could be trained immediately.

The Activity assumed that this model would be more sustainable; require fewer resources; and ensure continuity, ownership, and quality of Implanon insertion services at the HP level. However, there was limited evidence about the onsite Implanon training approach in terms of cost and time needed, quality of service delivery, and improved linkages between the HPs and HCs. The aim of this study was to assess the training standards were maintained, cost savings, and other potential benefits of onsite Implanon insertion training of HEWs compared with offsite training in four agrarian regions of USAID Transform: Primary Health Care Activity implementation.

### Program description: onsite Implanon insertion training of HEWs.

The Activity team adapted the training model for onsite Implanon insertion training of HEWs from *Programming for Training: A Resource Package for Trainers, Program Managers, and Supervisors of Reproductive Health and Family Planning Programs* [13], which illustrates how Implanon insertion training of HEWs, traditionally conducted offsite with groups of 20 to 25 HEWs, can be practiced at the PHCU level with a smaller number of HEWs using skilled providers as trainers onsite. The same national training curriculum was used for both the off-site and on-site trainings, which were both given for a total of 6 days and included 3 days of theoretical lectures and simulated skill practice on the Arm model, followed by 3 days of clinical attachment at healthcare facilities for skill practice. In this model, facility management and the FP service providers in the HC and HPs work together to plan trainings with inputs from partners and keeping in mind existing health-system resources. The Activity equipped HCs to provide trainings with minimal support from partners by forming a team of trainers comprised of competent clinical providers in the HCs; by providing Implanon insertion training materials; and by supporting organization, facilitation, and follow-up of the trainings.

**Fig. 1:**
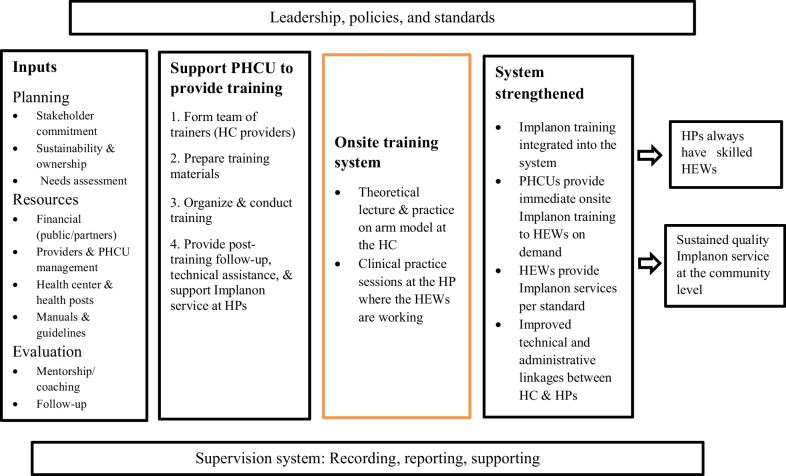
Model for onsite training of HEWs on Implanon insertion at the PHCU Level [13]

The model has the potential to improve the skills of HEWs; strengthen linkages between HCs and HPs and between HEWs and their catchment communities; and ensure the integration of Implanon insertion services, including follow-up and monitoring, into the health care system. The onsite approach allows HCs to offer timely training to their own HEWs on demand with minimal support from outside. As a result, HPs maintain a skilled workforce of HEWs, and ensure sustained, quality community-level Implanon insertion services (Fig. 1).

## Methods

### Study design and setting

The USAID Transform: Primary Health Care Activity, implemented in collaboration with the MoH of Ethiopia since 2017, has supported more than 400 woredas (districts), benefiting nearly 53 million people. The Activity strengthens the management and performance of Ethiopia’s national health system by improving service delivery process across the continuum of primary health care, improving household and community health practices and health care-seeking behaviors, and strengthening program learning to impact policy and activities related to the prevention of child and maternal morbidity and mortality. This study—part of an effort to support the Ethiopian government’s commitment to improve LARC-Implanon utilization within USAID Transform: Primary Health Care implementation regions—focused on onsite and offsite Implanon insertion trainings between 2017 and 2019. To introduce an alternative Implanon training approach to the conventional one and institutionalize in the PHCUs' health care system, Pathfinder supports both onsite and offsite training approaches at the same time in the project’s intervention districts and facilities. The data were collected in March 2020 using a mixed-method study design to assess the onsite Implanon Insertion training approach in four agrarian regions of Ethiopia: Oromia; Amhara; Southern Nations, Nationalities and Peoples’ (SNNP); and Tigray. During the time of data collection, Sidama and Southwest regions were part of SNNP; in this paper, the term “SNNP” is used to refer to these two regions. The source and study population of this analysis included, quantitative training information of onsite and offsite trainings and key informant interview of HEWs (trainees), trainers (health care providers), facility heads, and district-level FP heads and experts.

### Sample size and sampling

For the quantitative part, the study used training data of HEWs that attended offsite and onsite Implanon insertion training between 2017 and 2019 with the support of the USAID Transform: Primary Health Care Activity. The training data of 468 HEWs that attended onsite (234) and offsite (234) trainings were included in the study. Key informant interviews were also conducted with 20 purposively sampled individuals including four district health office heads and FP experts, six Implanon insertion trainers (both offsite and onsite), five health facility heads and five trained HEWs. The key informant interviewees were selected considering their knowledge and experience on both onsite and offsite trainings. Accordingly, they are all drawn from districts and/or facilities that implemented both onsite and offsite Implanon training with insight on both onsite and offsite training.

### Data collection

Quantitative information was gathered through Implanon insertion training registry books that were kept both on-site and off-site. Examples included the number of trained HEWs, women who received the service, model procedures, pre- and post-test results, and training costs and collected using a prepared questionnaire adopted from a training evaluation textbook and previous Implanon insertion training evaluation studies [5, 6]. The qualitative data collection tool was prepared in English and translated into regional working languages (Tigrigna, Amharic, and Oromipha). Data collectors with qualitative study experience and fluency in the regional working language carried out the key informant interviews using an interview guide. The instrument elicited detailed information about aspects of the onsite and offsite training approaches, including creation of local capacity, cost sharing, technical and administrative support, and engagement of HEWs and providers from HCs during and after the training, including trainee–client interaction.

Eight data collectors who were fluent in the regional working languages were selected for data collection, and four supervisors with experience in Implanon insertion training were selected for supervision. Training, consisting of mock interviews and practical exercises for both data collectors and supervisors, was conducted over a 2-day period in March 2020. The questionnaires were pretested and refined to ensure that they were clear and understandable for both the data collectors and respondents.

### Data processing and analysis

The researchers assessed the quality, accuracy, and completeness of the data using range plausibility and cross-validation checks. The quantitative data were entered into EPI-Data vs 3.02 for Windows and exported into SPSS vs 20 for further analysis. Data analysis consisted of descriptive statistics (table and graph) to summarize the quantitative data and a *t* test with *p* values < 0.05. By evaluating the expenses of the training approaches relative to the results, where results were solely financial in both training approaches, the investigator consider costing in their analysis methods. Qualitative key informant responses were audio-recorded, transcribed verbatim in local working languages (Amharic, Oromipha, and Tigrigna), and translated into English before analysis. Thematic analysis was used to interpret the data in three phases: preparation, team organization, and reporting of summary results. The first phase of analysis started with careful, iterative reading of the data for familiarity. In the organization phase, the first author read each transcript carefully and highlighted thematic text (words or phrases) that appeared to describe the phenomenon under study (time, sustainability, availability, usefulness). The highlighted theme text was openly and manually coded with descriptors. The other authors read the data to confirm the descriptive codes. The codes were revised, and the codes that emerged from the revision were jointly reviewed before they were integrated into the analysis. The other authors collaborated with the first author to review, discuss, and agree on the final code categories. The final analysis was summarized manually based on agreed-upon emerging themes.

## Results

The quantitative results shed light on maintained training quality, and efficiency of onsite Implanon insertion training for HEWs compared to offsite training.

### Maintain training standard and quality

A 6-day Implanon insertion training was provided onsite to a total of 234 HEWs and offsite to an additional 234 HEWs. Each training included 3 days of theoretical lecture and practice on a simulated arm model, and after completing the theoretical and practical sessions, three additional days of clinical service provision to actual clients. Onsite trainees did their clinical practice at their respective HPs. HEWs trained offsite did their clinical practice at HPs in the training area outside of their workplace, in both training approach attended by mentors.

There was no significant difference between the onsite and offsite trainees’ mean pre-test (61.4% and 61.6%) (*P* < 0.947) or post-test (86.4% and 84.4%) (*P* < 0.220) knowledge scores or the average number of simulated skill-practice sessions on arm models (4 and 3) (*P* < 0.202)*.* However, onsite training offered twice as much opportunity for clinical practice.

**Fig. 2:**
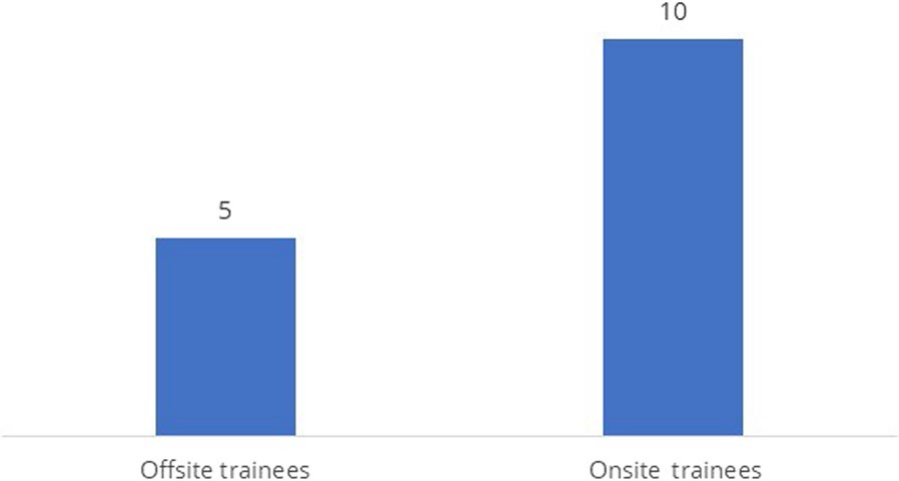
Average # Implanon insertions performed by trainees during 3-day clinical sessions

During the practical sessions, onsite trainees practiced clinical skills in their own HP by providing services for women mobilized according to eligibility criteria. The training facilitators followed the trainees with counseling and clinical skill checklists during the model and clinical practice sessions to ensure and maintain the quality of the training. The national skill requirement for Implanon training is to perform a minimum of five Implanon insertions during clinical attachment. During the onsite clinical attachment days, each HEW practiced Implanon insertion on an average of 10 clients; offsite trainees practiced on an average of 5 clients (Fig. 2). During onsite clinical practice, 2404 clients received Implanon insertion services, and during offsite clinical practice, 1080 clients received Implanon insertion services.

Of HEWs trained onsite, 150 (74%) performed an average of five or more Implanon insertions in the first 2 days of the clinical session, whereas none of the HEWs trained offsite performed five or more Implanon insertions in the first 2 days of the clinical session*.* HEWs trained onsite performed more than those trained offsite (*P* < 0.001), because HEWs in onsite training sessions had more clients with whom to practice (*P* < 0.001). In onsite training, post-training follow-up, support, and mentorship were integrated into the public-sector training system to ensure service quality and continuation and promote client satisfaction. The evidence shows that onsite training strengthened demand-creation activities, post-training follow-up and care, service registration, and reporting.

### Training cost

**Fig. 3:**
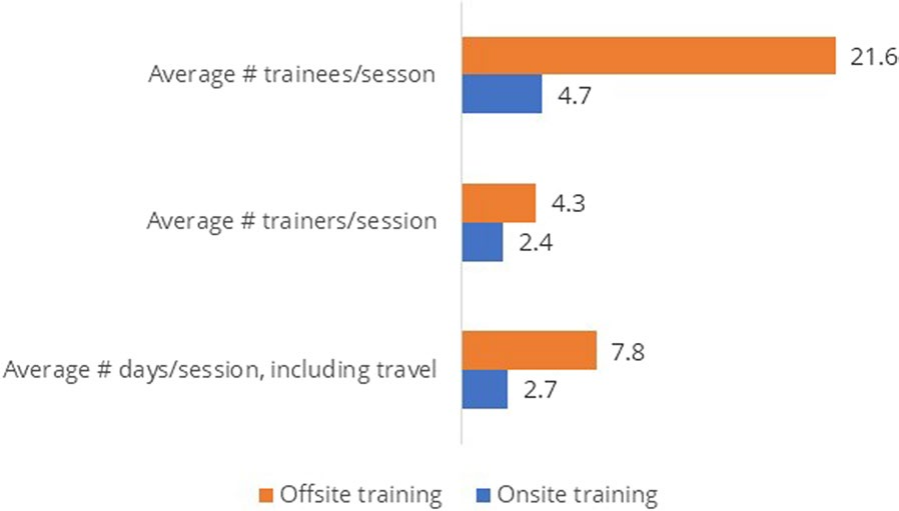
Average number of days, trainers, and trainees per training session

Onsite training saves both money and time. On average, the onsite training cost 1153 birr (37.2 USD) per participant, a savings of more than 75% (3563 birr = 114.9 USD) compared to the offsite training cost of 4716 birr (152.1 USD) per participant (*P* < 0.001) (Fig. 3). The training cost covered personnel expenses, such as trainee and trainer allowances, transportation, logistics and manuals, venue rental, paid working days for trainers and trainees, and refreshments. The average number of paid working hours trainers and trainees were away from their workplace during onsite training was 20.8 ± SD = 3.92; for offsite training, it was 62.2 ± SD = 3.38, a significant difference (*P* < 0.001). Moreover, HEWs who attended onsite training were away from their routine workplace for an average of 3 days compared to those who attended offsite training who were away for 8 days (*P* < 0.001) (Fig. 3). During the three clinical attachment days, onsite trainees were assigned to their respective HPs, which remained open for routine health services.

### Qualitative findings

The qualitative findings shed light on the quality, access, and sustainability of onsite Implanon insertion training for HEWs.

#### Quality, continuous care

Onsite training improves quality and continuity of care. All key informant interview respondents said that onsite training helped them to train HEWs immediately when needed, with fewer costs or interruptions to regular service delivery. Integrating training and post-training follow-up into the existing PHCU system allowed the trainers and trainees to work in a familiar environment with multiple opportunities to interact with clients from their catchment communities. One HEW shared, “During onsite training, the HEWs have multiple opportunities to communicate with clients, like during demand creation, insertion of Implanon, and follow-up. However, during offsite training, HEWs’ [have] contact with clients only during the training and not afterward.” The multiple opportunities for client–provider contact afforded by onsite training have improved and strengthened client–provider relationships and contributed to the continuum of FP care at HP levels.

In addition, all 20 sampled participants reported that the onsite training approach provided more opportunities for the HEWs to practice Implanon insertion, post-insertion care, and follow-up. A district health office FP expert praised the approach: “The HEWs have conducted the demand creation, scheduled clients for the clinical practical session, counselled, provided Implanon insertion service, and continued post-Implanon insertion care and follow-up after the training. This is different during the offsite approach, while all training activities are performed by other bodies, and after the training, HEWs have to return back to their respective HPs and [have] no chance to provide post-training care and follow-up."

#### Service availability and access

Onsite training ensures timely initiation and continuity of Implanon services. Most participants (96%) responded that the onsite training approach was preferable for rapid training of a small number of HEWs at the HC level to replace those who leave, rather than waiting for offsite training, which requires a larger number of HEWs to be scheduled, costs more, and requires more time. All key informants agreed offsite training keeps HEWs away from their workplaces longer than onsite training. One facility head said, “The offsite training is organized at a central level away from our workplace, requiring travel to the training place and more days away from the workplace, while during the on-site training, most HEWs join the same day of the training at the HC and [are] attached for the clinical practice at their respective HPs. This allows the HPs [to] remain open and provide health services."

All 20 key informants felt that the onsite training approach better facilitated pre-training activities, including needs identification and trainees selection, organization, and provision of training for HEWs. An Implanon trainer from a HC said, "We are able to use resources from the HC when there is a shortage of equipment and materials in the HPs. Being able to use a trainer from our own HC creates more responsibility to conduct and follow the progress of the training by the PHCU staff.” A PHCU head observed, “Because the trainers, trainees, and the clients are all in our sites, we can easily organize and conduct the training at any time, with minimal cost."

#### Public-sector ownership and sustainability

All 20 key informants reported that the onsite approach made it easier for the public sector to organize the pre-training, conduct the training, and provide immediate post-training follow-up and coaching with little or no support from partners or other entities. A trainer observed, “All the onsite training activities are organized and conducted by the HC with minimal partner support." A trained HEW said, “All activities from the pre-training to the post-training were organized, conducted, and coached [by] the HCs and HPs…. During the offsite training, the involvement of most HCs is limited only to the selection of HEWs for training." A PHCU head agreed: “In the offsite training approach, trainers and trainees come from different places, making it difficult for a HC to organize, conduct, and follow-up the training without partner support.”

Nineteen key informants reported that the onsite training strengthened linkages between the HP and the HC staff. One woreda expert said, "Because all the training activities from pre-training to post-training were organized and conducted by the PHCU staff with cooperation of partners, the trainers are from the HC, and the HEWs are from the same PHCU, and both already know each other…. In the offsite training approach, the trainer and the trainees are from different places or from different woredas, and their relation is only during the training and no relation after that."

## Discussion

Ethiopia’s introduction of offsite Implanon insertion training significantly increased access to LARCs, particularly Implanon contraceptive services, at the community level [4–6]. Despite this improvement, offsite IST has some limitations, such as cost, interruption of essential health care services at the HP level when HEWs are away from their sites to participate in training, and limited public-sector ability to train HEWs on demand, which can be resource-intensive and dependent on partners [10]. These limitations motivated the conceptualization of an alternative onsite training model for HEWs. This assessment shows that the onsite approach maintained the standard and quality of the training and minimizes HP-level service interruption. Similar studies have found that traditional training approaches that use extended, offsite, group-based workshops have limited effectiveness in improving and maintaining provider performance after training [10]. These studies signify the need to identify strategies to improve sustainability, effectiveness, and efficiency of IST [9], and recommend institutionalization of IST programs that use alternatives to offsite training [11].

Both the onsite and offsite approaches used the national curriculum to train HEWs on Implanon insertion. The findings of this study show no significant differences in pre- and post-test knowledge scores or in the number of insertions practiced on simulated arm models between HEWs who were trained onsite and those trained offsite. However, there was a significant difference in the average number of Implanon insertions performed on actual clients during clinical practice sessions: HEWs trained onsite practiced an average of 10 Implanon insertions, more than double that performed by HEWs trained offsite. This could be because the HEWs trained onsite did the demand creation themselves to generate enough clients for clinical practice. The fact that HEWs trained onsite performed an average of five or more Implanon insertions in the first 2 days of clinical attachment also demonstrates that the number of clinical attachment days for skill practice could be reduced from three to two. The MoH Implanon insertion training evaluation report indicates that offsite trainees were generally unable to practice on enough clients during clinical practice and not all trainees completed the required minimum of five insertions [4].

Organizing an offsite Implanon insertion training for HEWs requires many participants [3]. Many studies reported high turnover of health care providers, including HEWs, as a challenge for the health system in Ethiopia [14–16]. Sustaining the current fast pace of turnover and replacement training is a challenge, despite the work of the government of Ethiopia to recruit and deploy HEWs [17]. Onsite training allows the health system to train HEWs with minimum cost, on demand and immediately assign skilled HEWs to fill vacancies and ensure service continuity at the community level. This study shows that onsite training is appropriate to train a small number of HEWs—as few as one to three per session—because public HCs can organize, conduct, and follow the trainings independently by integrating them into existing services provided by the PHCU system.

Our study suggests that onsite training improves the linkages between HPs and HCs, including among staff. As part of the routine health service delivery system, health care providers from HCs are assigned to HPs and conduct regular visits to provide technical support and monitor health services in the HPs [18]. Onsite training creates an additional opportunity for the HEWs and HC staff to work together during the training and develop management and coordination skills, including organizing, conducting, following up, and reporting of training and service delivery. This creates a shared responsibility within the system and ensures sustainability of training and services at the program and community levels. In contrast, all offsite Implanon insertion trainings were done via public–partner collaboration, where partners were usually the main providers of the trainings, and this approach is not sustainable [4, 11]. A literature review found that regions and woredas with strong, well-coordinated PHCUs more effectively implemented the Health Extension Program and improved service utilization at the HP level [14].

The HEWs trained onsite had a closer relationship with their clients than those trained offsite, because there were multiple opportunities for interaction between HEWs and clients during onsite training, including information provision, demand creation, and clinical services. The interaction between HEWs and clients continued during post-insertion care and follow-up. In contrast, during offsite training, HEWs and clients interacted only once during the clinical session and thereafter returned to their respective HPs. A related study found that three-fourths of respondents who perceived a positive interpersonal relationship with HEWs they knew in person, preferred to receive health information or services from them [19]. Frequent interaction between HEWs and clients during onsite training yields more opportunity to ensure the FP continuum of care and improve service quality and acceptance.

### Limitations

This study's key findings demonstrated the value of an onsite training strategy. The limitations include, first, because there were not enough resources to construct a prototype onsite training program, the study did not cover many training sessions. Second, since this training paradigm is novel, there are fewer possibilities to compare the results of this study to those of comparable interventions and there is no comprehensive skill attainment evaluation to assess the "know-do gap.” Third, there is a possibility of bias in selectively reporting the strengths of onsite training among KII respondents, this study examined the perspectives of district and facilities health authorities, trainers, and trainees. We did not account for or control responder bias triggering factors in our analyses. For instance, some of the key informant participants preferred the onsite training approach, since the trainees would not be away from their facilities and would not interfere with their regular service. In addition, during their clinical attachment, the trainees would have numerous opportunities to communicate with their clients to generate demand, provide more insertion service, and give them the opportunity to follow their clients.

## Conclusion

Onsite Implanon insertion training is an appropriate approach to train a small number of HEWs on demand with minimum cost at the PHCU level. Onsite training minimizes the time HEWs spend away from their facilities, reducing days of service interruption. It improves linkages between linked HCs and HPs, including among staff; ensures a continuum of FP care; and strengthens interpersonal relationships between clients and service providers at the community level. In general, onsite training enhances local public-sector capacity and ownership, ensures sustainable LARC-Implanon insertion training programs for HEWs, service quality, and service continuity at the community level. In addition, the onsite training approach demonstrates the possibility of reducing the number of days of clinical training on actual clients from three to two, because HEWs were able to perform the minimum required five Implanon insertions during skill practice sessions in the first and second clinical training days during onsite trainings. Based on the study findings, we recommend that stakeholders consider onsite Implanon insertion training for HEWs as a viable alternative to offsite trainings, particularly in the context of rapid turnover of skilled health workers at the community level and scale the approach to other non-intervention PHCUs.

Our results highlighted key findings; however, some may not have included information regarding cost effectiveness, bias control, and comparability. Therefore, suggest undertaking additional research to solve all this study's flaws to revive an effective training technique.

## Data Availability

The data sets used and/or analyzed during the current study are available from the corresponding author upon reasonable request.

## Abbreviations

HC: Health center
HEW: Health extension worker
HP: Health post
FP: Family planning
FP/RH: Family planning and reproductive health
HSTP: Health Sector Transformation Plan
IST: In-service training
IUCD: Intrauterine contraceptive device
LARC: Long-acting reversible contraceptive
MOH: Ministry of Health
PHCU: Primary health care unit
USAID: United States Agency for International Development
